# Landscape structure and site characteristics influence whether the northern house martin *Delichon urbicum* occupies artificial nests

**DOI:** 10.1002/ece3.70261

**Published:** 2024-09-08

**Authors:** Gianna Allera, Ramona Julia Heim, Aline Förster, Wieland Heim

**Affiliations:** ^1^ Institute of Landscape Ecology University of Münster Munster Germany; ^2^ Department of Evolutionary Biology and Environmental Studies University of Zurich Zurich Switzerland; ^3^ NABU‐Naturschutzstation Münsterland e.V Münster Germany; ^4^ Swiss Ornithological Institute Sempach Switzerland; ^5^ Present address: Institute of Biology and Environmental Sciences University of Oldenburg Oldenburg Germany

**Keywords:** breeding, conservation, nestbox, nesting aid, occupation rate, swallow

## Abstract

Artificial nest sites can support populations of endangered species when they are correctly installed. Here we analysed the characteristics and conditions that determined whether the northern house martin *Delichon urbicum* occupied more than 300 artificial nests around the city of Münster, Germany. We found that artificial nest occupation rates were influenced by various environmental and temporal factors. Positive influences included a longer time since installation and, to a lesser extent, the number of artificial nests at the same site. Negative impacts were observed from higher proportions of sealed surface cover in the surrounding area and, to a lesser extent, southward exposure. The distance to the nearest water body and the number of occupied natural nests showed no significant effect. We compared our results with descriptive evidence from the grey literature and published reports, and we give recommendations for installing artificial house martin nests for conservation practitioners. Future studies should also investigate the potential negative effects of ‘dirt boards’ below the nests and of gaps between the roof and the artificial nests.

## Graphical abstract

Artificial nest sites can support populations of endangered species when they are correctly installed. We found that occupation rates of artificial House Martin nests were lower for nests with a higher proportion of sealed surface cover in the surrounding area but were positively affected by increasing time since installation and the number of artificial nests at the same site. We summarise the range‐wide evidence and compare it with our data to give the best possible recommendations for conservation practitioners on how to install artificial nests for this declining bird species.

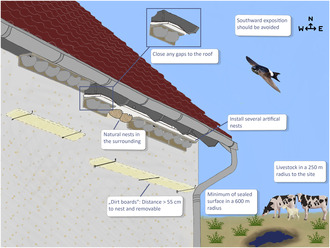

## 1 INTRODUCTION

Nestboxes and artificial nesting sites are widely used tools to support the conservation of certain cavity‐breeding species (e.g. Beyer & Goldingay, 2006; Libois et al., 2012; Newton, 1994), especially bird species that breed in or on anthropogenic structures since modern or renovated buildings offer few suitable nesting sites. Nestboxes have, therefore, become an important tool for the maintenance of city‐dwelling populations of species such as the peregrine (*Falco peregrinus*) and the common swift (*Apus apus*) (Altwegg et al., 2014; Dulisz et al., 2022; Sumasgutner et al., 2020). As such, understanding the factors that determine the successful occupation of artificial nesting sites is key for more widespread application of this conservation strategy by organisations, architects and building owners (Lambrechts et al., 2012; Schaub et al., 2016).

The northern house martin *Delichon urbicum* (hereafter: house martin) is a widely distributed Eurasian breeding bird that feeds on aerial insects and nests colonially on buildings. More rarely, it also nests on rock faces or other manmade structures (Glutz von Blotzheim & Bauer, 2001; Lind, 1960). House martins build their nests from collected mud and prefer to attach them under protruding roofs (Glutz von Blotzheim & Bauer, 2001). For this, they need open mud puddles or bodies of water from which they can collect nesting material. House martins, therefore, prefer nesting sites closest to sources of food and mud in areas with a high proportion of open space (Murgui, 2002). As colonial breeders, house martins are also known to prefer sites with old nests of conspecifics (Bell, 1983; Piersma, 2013).

The population of house martins is declining globally (BirdLife International, 2023), which is especially well documented in Europe (Keller et al., 2020). The reasons for this strong decline are diverse. Modern urban settlements often lack collection sites for nest building (Richarz & Homann, 2010), and increased surface sealing may cause mud puddles to disappear. Furthermore, house owners often prevent house martins from nesting on their façades to avoid the mess caused by droppings and the nest building (Gedeon et al., 2014). In addition, factors such as intensive agriculture, insecticides, habitat fragmentation and soil sealing may decrease the insect biomass, causing food shortages for house martins (Bryant, 1975; Møller, 2019).

One approach to support house martins is to provide them with artificial nests (Frederking et al., 2003), which can increase local populations (Menzel, 1996). Although this conservation technique is now widely applied, little evidence is available on where to best place the nests, so many artificial nests remain unoccupied (Piersma, 2013; Schmolz, 2020; Willi et al., 2011). Of the few studies that have examined this issue, a study in Switzerland found that higher occupation rates occurred for artificial nests that were placed (1) closer to natural nests, (2) oriented towards the south and (3) at farmhouses rather than other buildings (Meister & Ehrengruber, 2015); yet, strong statistical significance was only found for the condition in which natural nests were close by (Meister & Ehrengruber, 2015). In a recent study in Berlin, Germany, occupation rates for artificial nests were found to be greater for nests located higher above the ground (Dommaschke & Wardenburg, 2023). Although these studies offer important insights, most relevant information on this topic is hidden in grey literature or project reports (not peer‐reviewed, or in local languages), such that the effects of various factors on occupation rates have not been statistically tested.

The aims of our study were to (1) gain a better understanding of which characteristics and conditions, on the level of both the surrounding landscape and the actual site, determine house martins' successful occupation of artificial nests, which will help us (2) provide recommendations for how to best set them up.

## 2 MATERIALS AND METHODS

### 2.1 Fieldwork

**FIGURE 1:**
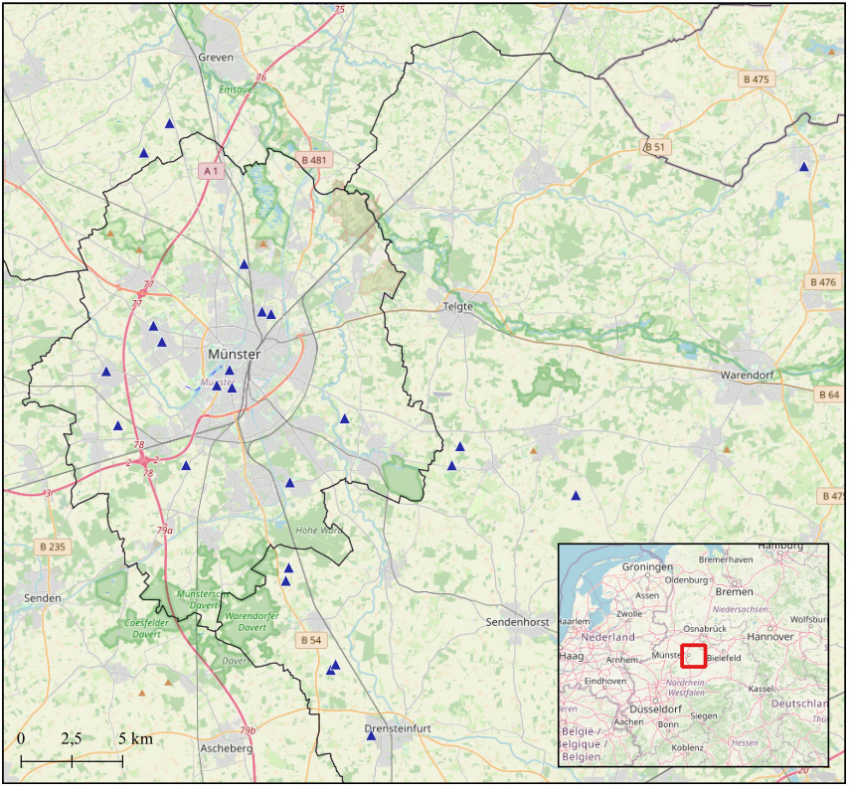
Location of house martin artificial nests (blue triangles) around the city of Münster (grey line) surveyed in this study. The inset shows the location of the study area in Germany. Background map: OSM Standard.

Our study took place around the city of Münster, North‐Rhine Westphalia, Germany. Here, the house martin is still a common and widespread species, but its population is strongly declining, for which the species is classified as endangered on the regional Red List of breeding birds (Gedeon et al., 2014; Grüneberg et al., 2016). We surveyed the occupancy of 311 artificial nests for house martins at 24 different locations (Figure 1).

All sites were visited once each during the three survey periods: 10–20 May 2021, 7–11 June 2021 and 5–10 July 2021. We counted a nest as being occupied if we had seen house martins flying in and out on at least two dates during the breeding season or if nestlings, feeding adults or fresh droppings were observed on at least one occasion. Observations were made all day for half an hour per site during mild and sunny weather.

**FIGURE 2:**
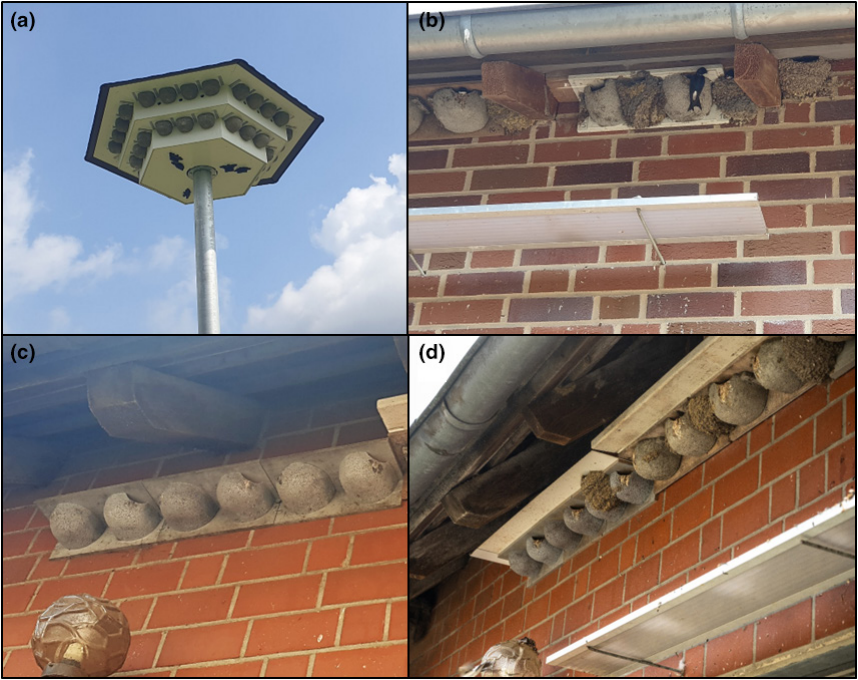
Differences in site factors of artificial house martin nests. (a) Swallow tower with multiple artificial nests, (b) dirt board below artificial and natural house martin nests, (c) roof gap over artificial nests, (d) gap over artificial nests was closed by installing a board above the nests. Photos by Gianna Allera.

The following variables were recorded for each artificial nest. First, the number of artificial nests in/on the same building was counted. Building type was also recorded using five categories: We differentiated between barns (barn ceiling in the interior, also called threshing floor), sheds (with or without livestock), residential houses and residential blocks as well as so‐called swallow towers (see Figure 2a). We also recorded the type of façade on which the nests were placed: We distinguished between sites with wooden beams, brick and rough facades. Exposure was measured in cardinal points using a digital compass. We also counted the number of occupied natural nests in a radius of about 250 m around the artificial nest, which is approximately the mean distance of house martins foraging trips (Glutz von Blotzheim, 1985). We calculated the degree of sealing for a square area of 1.44 km^2^ (length of each side = 1.2 km, based on a maximum foraging trip distance of ~600 m; Glutz von Blotzheim, 1985) in which the observation point was in the centre. In this square, 10 × 10 m grids were laid over the area, and the sealed grids were counted to determine the sealing percentage. We calculated the distance to bodies of water using satellite images on TIM‐Online (TIM‐Online, 2021) and the containing length measuring function. A wooden board (‘dirt board’) was installed below many of the artificial nests to protect the façade by catching any dirt (e.g. droppings or nest material) emerging from the nests (Figure 2b). We noted the occurrence of these dirt boards and estimated the distance between the nest and the board below. We also recorded whether other bird species had occupied the nest by looking for visible nesting material or incoming birds (e.g. house sparrow *Passer domesticus*). We also noted the time since installation (in years, 0 = before the breeding season of the same year, 1 = before the breeding season of the year before, and so on) and the presence of livestock (cows, horses, sheep, chickens or pigs). Finally, we also checked whether a gap was present between the nest and the roof (‘roof gap’, Figure 2c) or whether the nests were placed directly under the eave.

### 2.2 Data analysis

To analyse the impact of different characteristics and conditions on the occupancy of artificial nests, we had to exclude several variables from our statistical analysis, that were originally sampled during the study, because of an unbalanced sample size distribution between factor levels and collinearity. We therefore excluded the sampled data on building and facade type, other species, livestock, dirt board and roof gaps (Figure S1).

The final regression model included six continuous variables (time since installation, sealed surface cover, distance to water body, southward exposure, number of occupied natural nests, number of artificial nests) as independent variables and the occupancy of artificial nests (0 = not occupied, 1 = occupied) as dependent variable. For this, we transformed the directional variable exposition into a continuous variable, creating a linear scale where north is 0, south is 180 and both east and west are 90. The transformation involves adjusting values over 180 degrees, removing negative signs, and results in a range from 0 to 180.

We fitted a linear model in a Bayesian framework using the package brms (Bürkner, 2017, 2018, 2020) in R version 4.3.0 (R Core Team, 2023). We included the occupancy of an artificial nest as the dependent variable (0/1) and site as a random intercept. All independent variables were centred and scaled. We set family = ‘bernoulli’, iter = 4000 and warmup = 2000. We used default brms priors. We checked the model for multicollinearity (highest VIF = 1.36). We assessed the model assumptions using posterior predictive checks.

### 2.3 Literature review

We searched for literature using Google Scholar and the English and scientific species name plus one of the following search terms: nest box, nest, artificial nest, nesting aid or swallow tower. We also checked the references of these publications for further references. We searched for factors that were mentioned to affect artificial nest occupation and rated the provided evidence (strong, weak or poor). We considered evidence to be strong if the effect was statistically tested in at least one study, as weak when data were presented but not statistically tested and as poor when no data were presented (e.g. expert opinions).

## 3 RESULTS

We found that 78 artificial nests at seven locations were occupied by house martins, which corresponds to 25% of all nests. The percentages ranged from 22% to 86% of occupied artificial nests per location. No artificial nests were occupied at 17 locations. We divided our findings into variables that determine occupancy on the landscape level and variables of importance on the site level.

### 3.1 Landscape level

**FIGURE 3:**
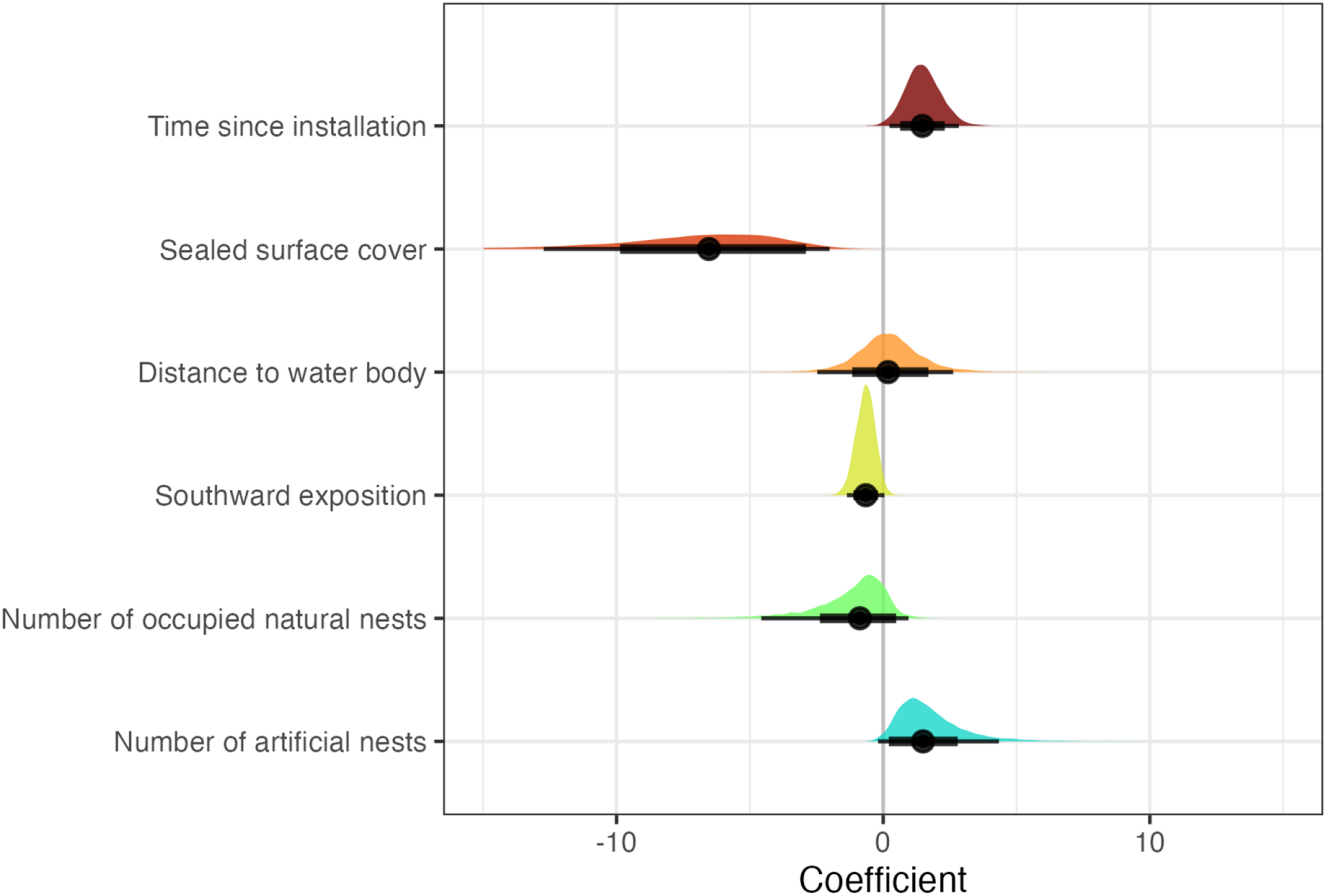
Estimated coefficients for each predictor variable. Black points represent the mean for each variable. The thin black line is the 95% credible interval, and the thick black line is the 80% credible interval. If the 95% credible interval lies completely to the left of 0 (e.g. for sealed surface cover), this indicates a strong negative effect of the variable on the occupancy of an artificial nest. The coloured area represents the full posterior distribution of the coefficient estimates for each variable. See also additional Figure S2.

Our model revealed a strong negative effect of sealed surface on the occupancy of artificial nests (Figure 3). On average, 25% of the area around the nests was sealed, with 9% at occupied nests and 31% at unoccupied nests. No significant effect was found for the distance to water bodies (Figure 3). The nearest body of water was, on average, 323 m away from the artificial nests, with a mean of 210 m for occupied and 357 m for unoccupied nests.

### 3.2 Site level

We found that the time since installation had a positive effect on occupancy (Figure 3). No nests that were newly installed in the year of study were occupied. Most nests in our study area (29.6%) were installed in the year of our survey (2021), 15.4% were installed 2 years earlier, 21.9% were installed 3 years earlier, 1.3% 4 years earlier, 24% 5 years earlier, 3.9% 8 years earlier and 2.6% 14 years earlier. The number of occupied natural nests at the same site had no significant impact, while the number of artificial nests was slightly positively linked to occupation (Figure 3). On average, 20.1 nests were installed at occupied sites, and 10.1 nests were installed at unoccupied sites. We furthermore found a negative effect of southward exposure on occupancy (Figure 3).

### 3.3 Additional observations

Due to collinearity and limited sample size, we could not include all other variables in our model. Here we briefly summarise our anecdotal observations: Livestock was present at all sites of occupied artificial nests (Figure S1), whereas no nests were occupied at sites without livestock in the surroundings (*n* = 10 sites). Natural nests were present at 15 locations, of which 11 locations had also occupied natural nests. On average, 6.4 intact natural nests at the same building or yard were found by occupied artificial nests, versus 5.8 natural nests by unoccupied artificial nests. At sites with active natural nests (*n* = 11), an average of 27% of the artificial nests were occupied, whereas if there were none (*n* = 13), only 4% of the artificial nests were occupied (Figure S1). Regarding the type of building, we found that 13% of the artificial nests were occupied at residential houses, 39% at sheds where livestock were kept and 44% at barns (Figure S1). No artificial nests were occupied in swallow towers (*n* = 2 sites) or at residential blocks (*n* = 5 sites). Regarding the façade type, 50% of the artificial nests were occupied at wooden facades (*n* = 8 sites), 20% at brick facades (*n* = 15 sites) and 0% at rough facades (*n* = 1 sites). No artificial nests were occupied if there was a roof gap (*n* = 6 sites, 76 artificial nests); only artificial nests that were placed directly under the eave were occupied (Figure S1). If a dirt board was present (*n* = 4 sites, 72 nests), artificial nests were only occupied if the distance between the board and the nest exceeded 55 cm. There was only one site where nests with a dirt board were occupied. However, at this site, the dirt board was only installed after the house martins had settled in. When other bird species had already occupied the artificial nests, house martins did not occupy them (Figure S1); we had four cases where artificial nests were occupied by house sparrows and one case by a Eurasian blue tit (*Cyanistes caeruleus*).

## 4 DISCUSSION

We found that a quarter of the artificial nests in our study were occupied by house martins. Based on our data, we were able to determine variables that impact their occupancy.

### 4.1 Landscape level

The percentage of sealed surface in the surrounding area was the strongest predictor for occupancy of artificial nests in our model. The less surface area that was sealed, the higher the probability that artificial nests were occupied. House martins' preference for open areas was found previously in studies of natural house martin colonies and has been explained by differences in food abundance (Donnerbaum & Wichmann, 2006; Murgui, 2002). A more natural environment provides more flying insect biomass and, therefore, offers better chances of successfully raising young (Bryant, 1975).

We found no significant effect of the distance to water bodies on occupancy. This was unexpected, as water bodies are not only important for foraging but also for collecting nesting material to construct natural nests (Bryant, 1975; Glutz von Blotzheim & Bauer, 2001). Natural colonies are also found more often in areas with water bodies close by (Donnerbaum & Wichmann, 2006; Lind, 1960). Possibly, very small waterbodies not included in our analysis (such as puddles) might be of greater importance.

### 4.2 Site level

We found a positive relation between occupation and time since artificial nest installation, with no occupancy in the first year. This could be due to house martins' site faithfulness, as demonstrated by ring recoveries (Fischer et al., 2023; Szép et al., 2017): If possible, adults prefer to return to their former colonies instead of colonising new buildings. However, we cannot confirm with our data whether it is the recent installation that it is the cause for nests being unoccupied or the fact that the nests have been placed in sub‐optimal locations. Future research should consider installing new nests at both established and potential colony sites to better understand factors affecting artificial nest occupancy.

Our model suggested that the number of artificial nests at a given site has a weak positive link to the occupation rate. The more artificial nests that were installed, the higher the likelihood of nests being occupied. However, this relationship is likely more complex. While we observed that sites with more artificial nests tended to have higher occupation rates, we cannot conclude a direct causal relationship. Several factors may contribute to this association. First, sites with more nests may inherently be of higher quality, attracting more birds regardless of nest numbers. Second, the correlation could stem from adding artificial nests to already established sites, where occupation is more likely. Previous research has noted that once house martins begin breeding in artificial nests at a specific location, the remaining artificial nests often become occupied quickly (Löhrl, 1973 in (Menzel, 1996)). Our observations of multiple occupied artificial nests at seven sites support this idea. However, it is crucial to note that simply increasing the number of artificial nests may not lead to occupation at unsuitable sites. The relationship between artificial nest number and occupation rates is likely influenced by a combination of factors, including site quality and existing colony presence. A study in Berlin, Germany found a significant negative effect of the number of artificial nests in a 1 m radius on the occupation rate (Dommaschke & Wardenburg, 2023).

Our model suggests a weak negative effect of southward exposure. This is fortified by observations from Berlin, Germany (Dommaschke & Wardenburg, 2023), but it contradicts a study in Switzerland, where house martins were found to prefer southward exposure, although this finding was not statistically significant (Meister & Ehrengruber, 2015). Local climatic conditions might cause differences in exposure preferences. We argue that the exposure direction is generally less important for occupation as long as the nesting site can be approached freely by the birds and they are somewhat shielded from the effects of solar radiation and severe weather (Bell, 1983 in (Fally, 1989)).

We found no significant effect of the number of occupied natural nests on the occupation rate which indicates that it is less important how many natural nests are present. However, natural nests were present at all study sites where artificial nests were occupied. Many sources recommend installing artificial nests at sites where natural nests are present (Carels, 2015; Menzel, 1996). Yet, the presence of natural nests does not necessarily lead to occupancy, as has been shown elsewhere as well (Piersma, 2013). It has to be noted that artificial nests are also intended as a conservation measure to establish new house martin colonies and not always to support existing ones. Our results demonstrate the attractiveness of artificial nests and, thereby, support the idea that artificial nests might be useful to guide existing colonies away from locations where they are not wanted and towards nearby locations where they are allowed to breed (Menzel, 1996).

### 4.3 Additional observations

Several variables might be of further relevance for the occupation of artificial house martin nests, but our limited data did not allow for the inclusion of those into our model. Here we briefly discuss our anecdotal observations in the light of the existing (grey) literature (see also summary in Table 1).

**TABLE 1:** Factors that may affect the occupancy of artificial nests by house martins, the direction of the effect and the available evidence.

Factor	Effect	Evidence	Sources
Time since installation	Positive	Strong	This study
Sealed surface cover	Negative	Strong	This study, Richarz & Homann, 2010
Distance to water	No	Strong	This study
Southward exposure	Negative	Strong	Dommaschke & Wardenburg, 2023, this study, Stevens, 2021
Southward exposure	Positive	Weak	Meister & Ehrengruber, 2015
N artificial nests B	Positive	Strong	This study, Schwegler, 2023
N artificial nests B	No	Weak	Meister & Ehrengruber, 2015
N artificial nests 1 m	Negative	Strong	Dommaschke & Wardenburg, 2023
Height above ground	Positive	Strong	Dommaschke & Wardenburg, 2023, Stevens, 2021
Height (relative)	Positive	Strong	Elle & Lanfer, 2023
Building type: Farms	Positive	Weak	Meister & Ehrengruber, 2015
Building type: Swallow towers	Negative	Weak	Carels, 2015, Dommaschke & Wardenburg, 2023, this study
Façade type	No	Weak	Carels, 2015, Meister & Ehrengruber, 2015, this study
Façade type: wooden	Positive	Poor	Schwegler, 2023
Façade type: bright	Positive	Poor	Schwegler, 2023
Other species	Negative	Weak	Hoffmann & Michler, 2015, this study
Livestock	Positive	Weak	Meister & Ehrengruber, 2015, this study
N Livestock	No	Strong	Willi et al., 2011
Livestock duration	No	Strong	Willi et al., 2011
Dirt board	No	Strong	Dommaschke & Wardenburg, 2023, this study
Distance to dirt board	Positive	Weak	This study, Blischke & Trapp, 2017, Stevens, 2021
Roof gap	Negative	Weak	This study, Stevens, 2021
Natural nests	Positive	Strong	Elle & Lanfer, 2023, Carels, 2015, Meister & Ehrengruber, 2015, this study
Occupied nests	No	Strong	This study

*Note*: The direction of the effect was defined as positive if occupancy increases and as negative if occupancy decreases with increasing values of the factor. We considered evidence to be strong if the effect was statistically tested in at least one study, as weak when data were presented but not statistically tested and as poor when no data were presented (e.g. expert opinions). The study with the strongest evidence is listed first. Abbreviations are as follows: N artificial nests B = number of artificial nests at the same building; N artificial nest 1 m = number of artificial nests in 1 m radius around the nest; Height (relative) = relative height of the artificial nests in relation to height of roof overhang of surrounding buildings; N livestock = number of livestock; Livestock duration = the duration that livestock has been present in the stable; Occupied nests = number of occupied natural nests. For the definitions of the factors, please refer to the Section 2.

The presence of livestock in the surroundings may have a positive effect on the occupancy of artificial nests. In the Swiss study, only one farm with livestock was included, and here all artificial nests were occupied (Meister & Ehrengruber, 2015). This could be explained by a better food supply, as large herbivores and their droppings increase insect abundance, which has been linked to the breeding success of house martins (Bryant, 1975; Willi et al., 2011). However, other studies have also found that house martins occupy artificial nests at sites without livestock (Meister & Ehrengruber, 2015).

The type of building may influence occupancy as well. A higher occupancy rate at farm buildings was found in Switzerland, but artificial nests can successfully be installed at a wide range of buildings (Meister & Ehrengruber, 2015). The house martins' preference for farm buildings such as sheds and barns is again likely associated with food availability, as the surroundings of farms often provide higher abundances of flying insects (see above).

Another building type, namely swallow towers, have increasingly been installed for house martins in Europe, in many cases as a conservation compensation measure when old buildings that had supported a house martin colony were demolished. Several studies have reported that such structures are not or are only very rarely occupied by house martins (Carels, 2015; Dommaschke & Wardenburg, 2023; Meister & Ehrengruber, 2015). However, there are conditions under which these towers are occupied, e.g. when the height of the artificial nests at the swallow tower is greater than the height of the roof overhang of surrounding buildings (Elle & Lanfer, 2023). Playing back recordings of house martin calls may further help to increase their occupation rates (Gabay, 2022). But, given the high costs of installing such towers and the overall low chance of occupation by house martins, we recommend installing individual artificial nests at existing buildings, at least until the conditions under which these towers are efficient become more clear (see also (Carels, 2015)).

The façade type might also affect the occupation rate, as very smooth surfaces hinder the construction of natural nests (Glutz von Blotzheim & Bauer, 2001). Artificial nests might allow the colonisation of façades that are otherwise unsuitable for natural nests, such as PVC façades (Kettel et al., 2021).

In our study, no artificial nests were occupied if there was a gap between the nests and the roof. We assume that house martins avoid artificial nests with such gaps, perhaps because such nests are less shielded from weather or predators. At one location in our study area, such a gap was closed by installing a board in the eave above the nests, and the nests were occupied thereafter. This simple measure could significantly improve the occupation rate of artificial nests.

At some of our study sites, dirt boards were placed under the nests to prevent staining of the façade and the ground below. Our limited and unbalanced data did not allow to understand the effect of these dirt boards. Two sites had dirt boards less than 55 cm below the artificial nests, and these were unoccupied—however, this might be explained by the high percentage of sealed surface cover or the rather recent installation. At the one site where artificial nests were occupied above a dirt board, the distance between the boards and the nests was greater than 55 cm, and the boards were installed after occupation. Other studies have claimed that artificial nests with dirt boards that are placed closer than 50 cm to the nest are probably avoided (Blischke & Trapp, 2017; Richarz & Homann, 2010). House martins require free arrival and departure (Richarz & Homann, 2010), and natural nests are often built where the flight space is largest (Lind, 1960). Further, we observed that the dirt boards were used as perches by house sparrows, which might disturb the house martins. In addition, dirt boards can make it easier for predators to access the nests (Richarz & Homann, 2010). Notably, dirt boards were generally only installed on residential houses in urban areas, where cleanliness and appearance of the façade seem to be more important. On farm buildings in agricultural areas, the potential staining of house walls or floors is likely less of a concern, and dirt boards were only rarely installed. Therefore, the use of dirt boards is strongly connected to other factors, such as building type, the occurrence of livestock and the surrounding landscape, which makes it impossible to show the true effect of dirt boards with our dataset.

If other species have occupied the artificial nests, house martins are not able to use those. In a study in Switzerland, occupation by bats and wasps was found to prevent house martins from using artificial nests (Hoffmann & Michler, 2015). House sparrows are also well known to usurp house martins' nests (Lind, 1962), which can lead to the displacement of colonies (Tryjanowski & Kuczynski, 1999) even though house martins have been observed to cooperatively defend their nesting sites (Ieziekel & Yosef, 2020).

One factor not included in our study was the installation height above the ground. A recent study showed that the occupation probability increases with height (Dommaschke & Wardenburg, 2023).

Our study focussed only on the occupation of artificial nests, but not on breeding success. However, it can be expected that breeding success might be higher in artificial nests since the number of eggs correlates negatively with time that a pair needs to construct the nest (Kettel et al., 2021; Menzel, 1996). In artificial nests, no time is needed for nest construction, for which the birds can lay eggs immediately, which leads to larger clutches and, possibly, higher breeding output. Furthermore, nest collapse has been found to be the most common cause of breeding failure in natural nests (Kettel et al., 2021), while very unlikely in artificial nests. However, Piersma (2013) observed that breeding started later in artificial nests compared to old natural nests, which may negatively affect breeding success. Future studies should compare the breeding output of house martins breeding in artificial versus natural nests. Also worth investigating might be the difference in parasite load in natural vs. artificial nests.

### 4.4 Recommendations for conservation practice

**FIGURE 4:**
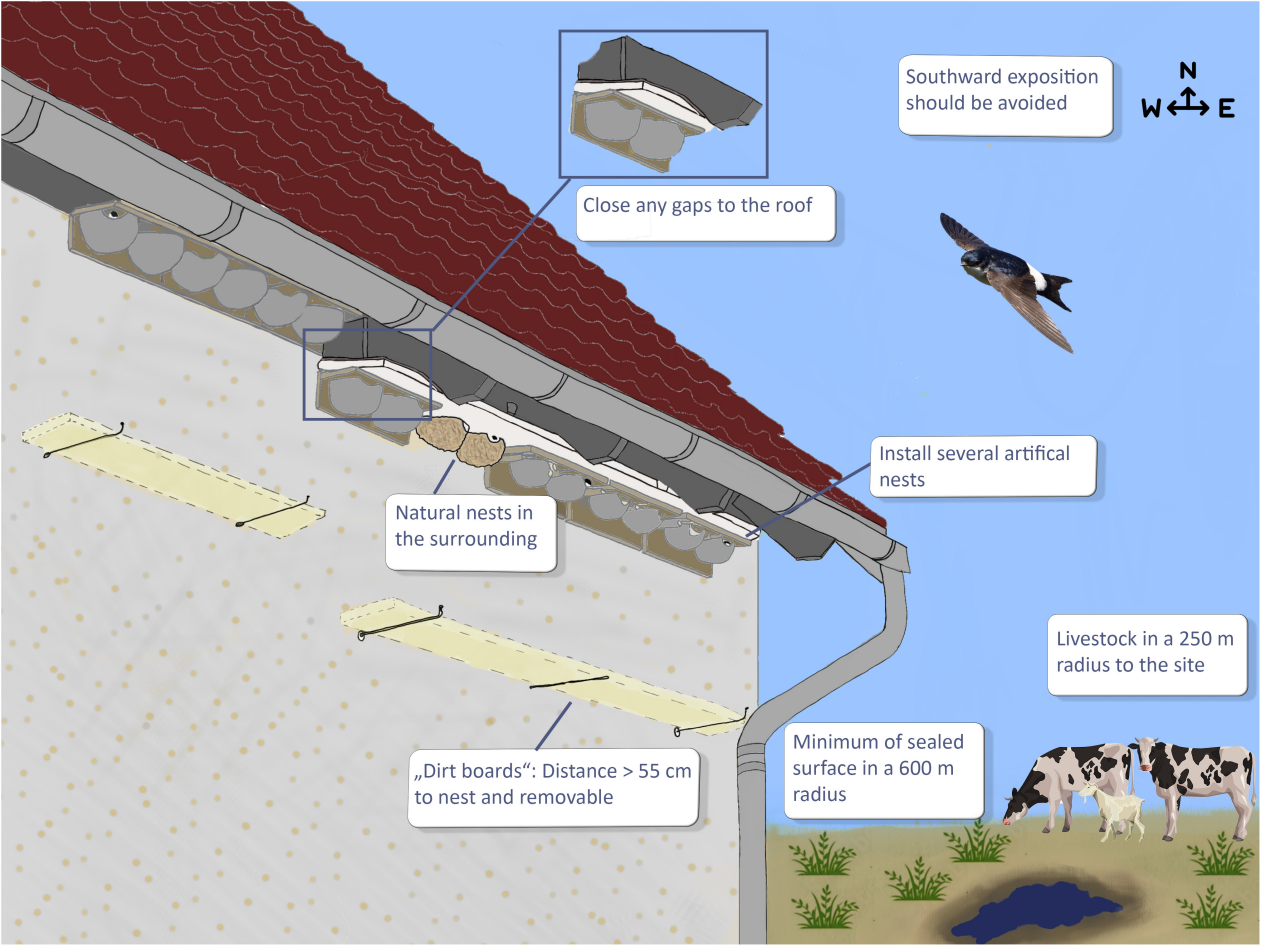
Illustration of how to place artificial nests to increase their occupation rate by house martins, based on the results of this study and a review of existing grey literature. Artwork by Gianna Allera.

In order to counteract the decline of house martins, artificial nests can be a helpful tool. Based on our data and the available grey literature (see summary in Table 1, Figure 4), we recommend using artificial nests at locations that provide a suitable habitat in the surrounding area, which can be determined by (1) a minimum of sealed surfaces, (2) proximity to water bodies (preferably closer than 300 m to the site) and (3) the presence of livestock in a 250 m radius to the site. The following site characteristics are assumed to increase the occupation rate of artificial house martin nests: Installing artificial nests (1) at sites with occupied natural house martin nests, (2) at farm buildings and (3) at locations where there is no gap between the nest and the roof. Furthermore, we recommend installing several artificial house martin nests at the same building, with no more than two other nests in a radius of 1 m and as high up as possible. If possible, southward exposure should be avoided. Dirt boards might be important to encourage building owners to allow house martins on their property, but these boards should be placed at distances greater than 55 cm below the nests and possibly only after the house martins have occupied the nests. Wherever possible, we recommend placing artificial nests at existing buildings instead of installing so‐called swallow towers. Furthermore, we urge for patience, as it can take years for artificial nests to become occupied.

## 5 CONCLUSIONS

Financial support for conservation measures is limited, and funds should be spent in the best possible way. We provide cautious recommendations on how to best place artificial nests for house martins based on our limited data and descriptive evidence from (mostly grey) literature. Our analysis was hampered by the fact that many of the variables were unbalanced and strongly correlated in our dataset, such that only a handful of the surveyed variables could be included in our statistical analysis. Future studies should investigate larger samples from different study areas and preferably use a more experimental setup to detect the importance of the factors that showed potential effects in our study, such as the roof gap. This would be especially relevant for variables that will increase the likelihood of building owners to put up artificial nests, such as the existence of dirt boards and the distance they could have to the nests without negatively affecting occupation rate.

## AUTHOR CONTRIBUTIONS

**Gianna Allera:** Conceptualization (supporting); formal analysis (equal); investigation (lead); visualization (equal); writing – original draft (lead); writing – review & editing (equal). **Ramona Julia Heim:** Formal analysis (equal); visualization (equal); writing – review & editing (equal). **Aline Förster:** Conceptualization (lead); investigation (supporting); writing – original draft (supporting); writing – review & editing (supporting). **Wieland Heim:** Conceptualization (lead); formal analysis (supporting); writing – original draft (supporting); writing – review & editing (lead).

## CONFLICT OF INTEREST STATEMENT

The authors declare no competing interests.

## Supporting information


Figures S1–S2.

## Data Availability

All data and R scripts are publicly available on Dryad (https://datadryad.org/stash/share/qU9M2sow_3kS_Yc‐Jpbxo6QRkKw‐UwW5vGC8GXdDHuM).
