# Supplementary material for: Landscape structure and site characteristics influence whether the northern house martin Delichon urbicum occupies artificial nests
Source: Ecol Evol. 2024 Sep 8;14(9):e70261. doi: 10.1002/ece3.70261 (PMC11381187; doi:10.1002/ece3.70261)
Supplement: Supplementary file 1 — Figures S1–S2. [file ECE3-14-e70261-s001.docx]

**Supplementary information**


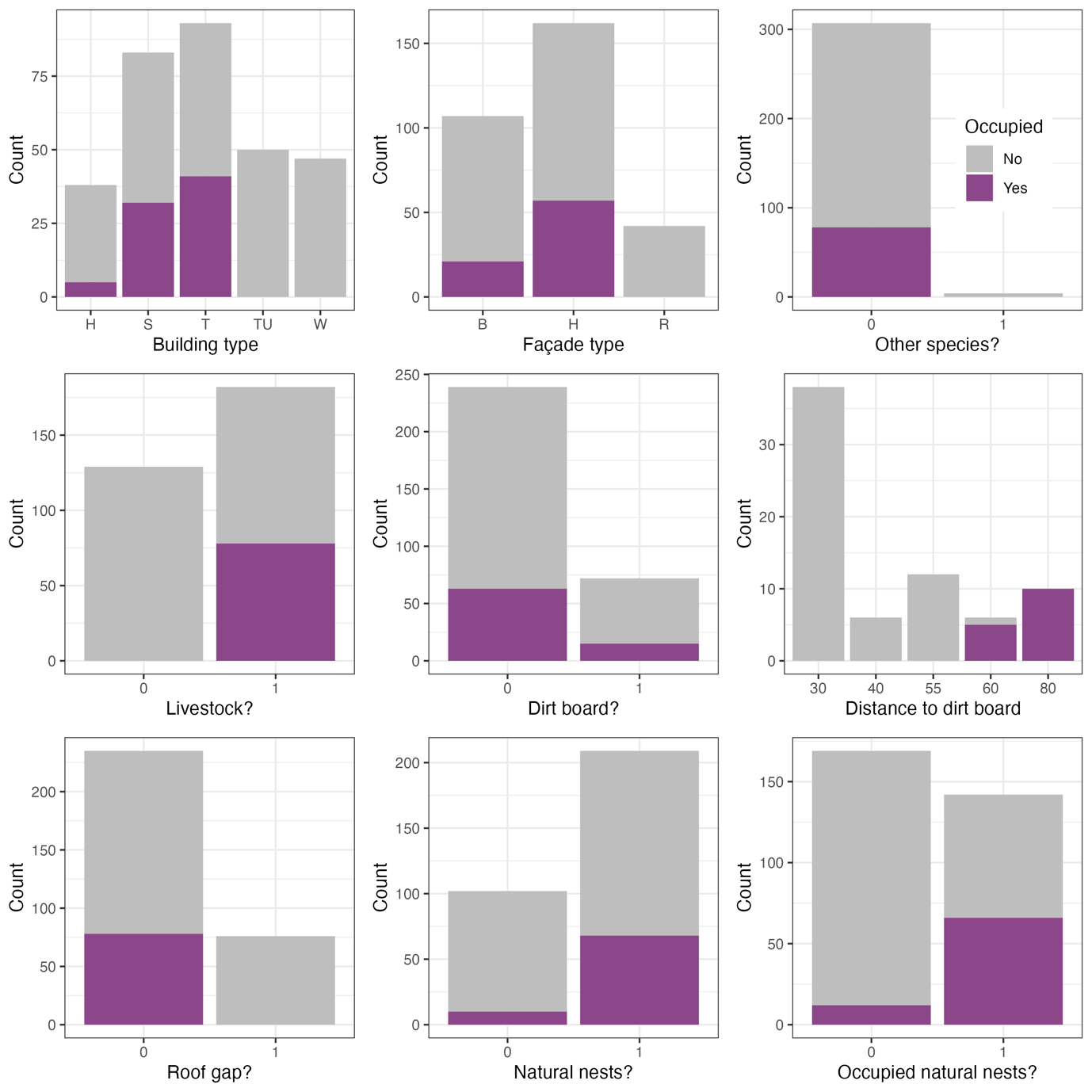


**Figure S1:** Count of occupied and unoccupied nests of the common house martin in relation to various building and landscape factors (grey = not occupied, orchid = occupied). The categories for building type are T = barn, S = shed, H = residential house, W = residential block, TU = swallow tower. Abbreviations for façade type are H = wooden, B = brick, R = rough. The distance to the dirt board was estimated in centimetres. For the variables with only two levels, 0 = no and 1 = yes.


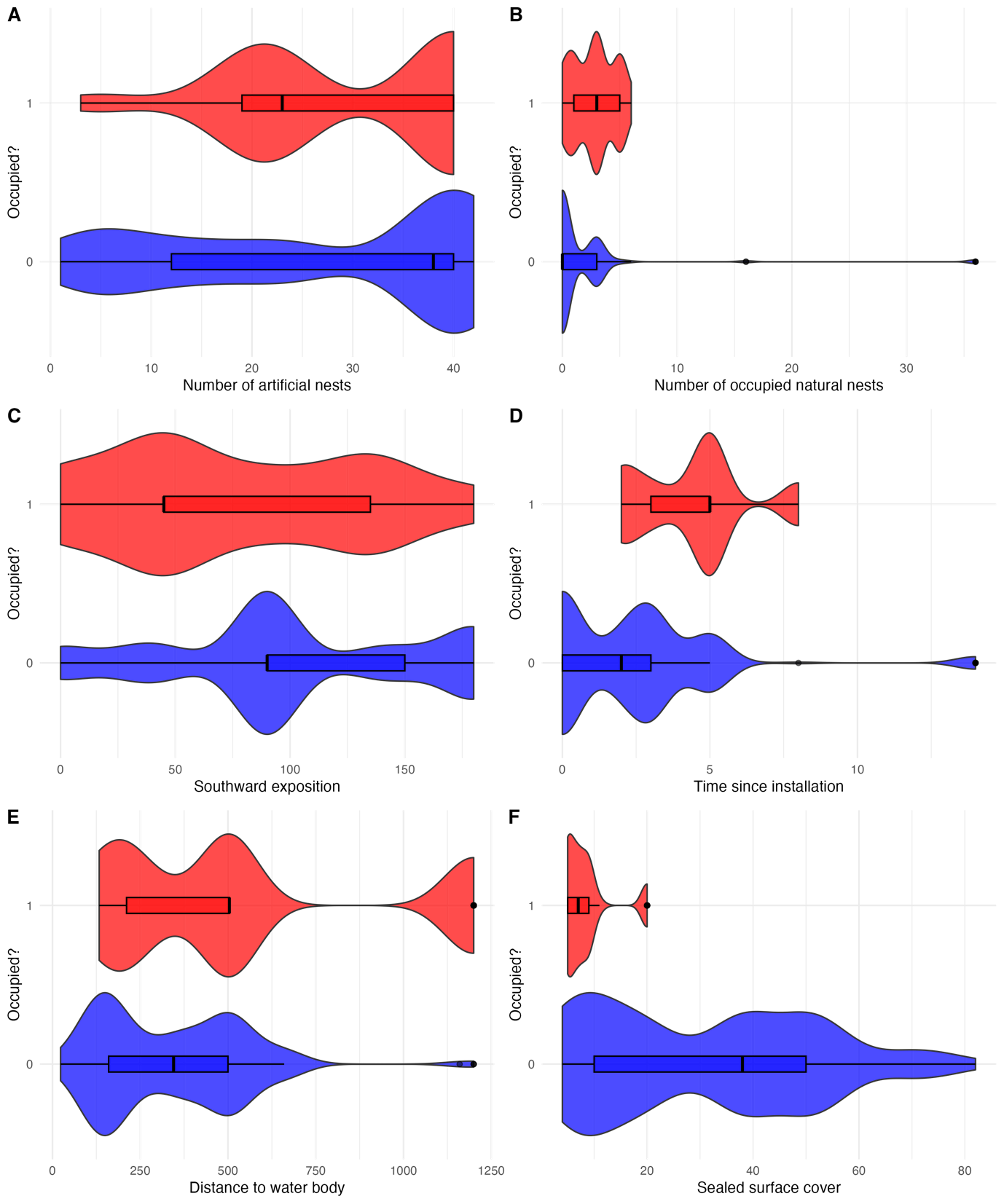


**Figure S2:** Violin plots showing the density of the six independent variables for the occupancy of artificial nests.
